# Longitudinal relationships among physical activity, internet gaming disorder, and insomnia: a cross-lagged panel analysis

**DOI:** 10.3389/fpsyt.2026.1903076

**Published:** 2026-07-08

**Authors:** Huijie Wang, Tiantian Cao, Lin Luo

**Affiliations:** 1 School of Physical Education, Jiangxi Normal University, Nanchang, Jiangxi, China; 2 Shangrao High Speed Railway Economic Experimental Zone Middle School, Shangrao, Jiangxi, China

**Keywords:** cross-lagged panel model, insomnia, internet gaming disorder, longitudinal mediation effect, physical activity

## Abstract

**Background:**

Internet gaming disorder (IGD) and insomnia are highly prevalent and comorbid public health issues among college students. While physical activity (PA) is a recognized protective factor, the longitudinal dynamic interactive mechanisms among PA, IGD, and insomnia remain unclear. This study investigated the longitudinal bidirectional relationships among these variables, focusing on the mediating role of IGD between PA and insomnia.

**Methods:**

A three-wave longitudinal study (3-month intervals) was conducted with 475 Chinese college students (aged 18.43 ± 1.71). Assessments included the Physical Activity Rating Scale-3, the Internet Gaming Disorder Checklist, and the Athens Insomnia Scale. A cross-lagged panel model (CLPM) was constructed, and mediation was tested using the bias-corrected Bootstrap method.

**Results:**

CLPM analysis revealed that: (1) PA was significantly and negatively correlated with subsequent IGD (T1→T2: β = −0.113, p < 0.001; T2→T3: β = −0.081, p = 0.029) and insomnia (T1→T2: β = −0.098, p = 0.003; T2→T3: β = −0.089, p = 0.003), whereas later-stage IGD was significantly and inversely correlated with subsequent PA (T2→T3: β = −0.123, p = 0.003), but the earlier-stage correlation was not significant (T1→T2: β= −0.052, p=0.175); (2) IGD was consistently and positively correlated with subsequent insomnia (T1→T2: β = 0.131, p=0.010; T2→T3: β = 0.137, p = 0.005), whereas insomnia was positively correlated with subsequent IGD only in the early stage (T1→T2: β = 0.132, p = 0.001), with the later-stage correlation being non-significant (T2→T3: β = 0.103, p = 0.055); (3) insomnia was not significantly correlated with subsequent PA (T1→T2: β = −0.030, p = 0.408; T2→T3: β = −0.065, p = 0.151); (4) T1 PA predicted lower T3 insomnia both directly and indirectly via lower T2 IGD (indirect effect = −0.005, 95% CI [−0.010, −0.002]).

**Conclusion:**

longitudinal associations exist among PA, IGD, and insomnia. PA emerges as a key prospective protective factor, and IGD plays a significant longitudinal mediating role in the association between PA and sleep, providing practical insights for preventing IGD and insomnia through increased physical activity.

## 1 Introduction

With the rapid development of digital technology, online gaming has become an integral part of college students’ daily entertainment and social life (1). However, Internet Gaming Disorder (IGD), resulting from excessive gaming behavior, has gradually emerged as a major concern in the global public health field (2). IGD is listed in the Diagnostic and Statistical Manual of Mental Disorders, 5th Edition (DSM-5) as a clinical condition requiring further research and has been formally included in the category of behavioral addictions in the International Classification of Diseases, 11th Revision (ICD-11) (3). University students are in a critical developmental stage of transition from adolescence to adulthood and face multiple challenges, including academic pressure, interpersonal adaptation, and career planning; consequently, they are more prone to exhibiting high levels of gaming dependency (2, 4). At the same time, insomnia symptoms are equally prevalent among college students. A large body of research indicates that the incidence of insomnia among college students is significantly higher than that of the general adult population and shows a persistent upward trend (5, 6). It is worth noting that IGD and insomnia have a high comorbidity rate; individuals with IGD often experience more severe sleep problems, while those with chronic insomnia are also more likely to exhibit problematic gaming behavior (7).

Unlike the widely used concept of “sleep quality,” insomnia is a clinically significant cluster of sleep disorder symptoms. Its core characteristics include not only difficulty falling asleep, difficulty maintaining sleep, or early awakening, but, more importantly, a persistent state of hyperarousal and cognitive-emotional processes such as cognitive rumination (8, 9). In recent years, physical activity (PA) has been recognized as a safe, low-cost, and easily scalable non-pharmacological intervention and an important protective factor for promoting mental health and improving sleep (10, 11). Moreover, it also influences the academic performance of adolescents (12). A growing body of research has found that regular physical activity not only improves sleep quality but may also reduce the risk of behavioral addictions, including problematic internet use and IGD (13, 14). However, most current studies on the relationship among PA, IGD, and insomnia are limited to pairwise correlation analyses. There remains a lack of a systematic understanding of the dynamic, interactive mechanisms among these three factors over time. Therefore, it is necessary to explore the processes of mutual influence among them from a longitudinal developmental perspective.

First, PA, serving as an important protective factor, may have a bidirectional association with IGD. Cross-sectional studies have shown that internet gaming addiction is negatively correlated with physical activity (15, 16). A prospective cohort study in South Korea confirmed that higher levels of physical activity are typically associated with milder symptoms of gaming addiction. In contrast, the incidence of internet addiction is significantly higher among those who lack exercise compared to those who exercise regularly (17). Furthermore, similar to other sedentary behaviors, online gaming not only displaces time allocated to physical activity and contributes to a sedentary lifestyle (18), but also, due to a lack of regular exercise habits, leads individuals to spend more time on online gaming (19). Conversely, increasing physical activity not only reduces the risk of IGD by decreasing gaming time (20), but also effectively alleviates the severity of gaming disorder and anxiety levels (21). Relevant systematic reviews further confirm the positive role of physical activity in reducing Internet gaming disorder and improving emotional well-being (22). Therefore, physical activity and Internet gaming addiction may also influence each other over time.

Similarly, a bidirectional association may exist between PA and insomnia. Furthermore, this relationship has been empirically supported by a large-scale longitudinal study (23). From a protective perspective, physical activity can improve insomnia through various physiological mechanisms. Physiologically, physical activity increases the body’s energy expenditure, thereby enhancing sleep drive; additionally, the rise in body temperature following exercise and the subsequent cooling process facilitate sleep onset (24). Furthermore, empirical studies have demonstrated that physical activity is negatively correlated with rumination and insomnia (25, 26). Additionally, intervention studies have further shown that exercise can effectively alleviate cognitive rumination and emotional hyperarousal in individuals with insomnia, while also reducing depressive symptoms (27). A recent meta-analysis has further corroborated these findings (28). However, insomnia may also negatively impact physical activity levels. Chronic sleep disturbances can lead to daytime fatigue, reduced energy, and diminished motivation to exercise, making individuals more prone to sedentary behavior and thereby reducing the likelihood of subsequent physical activity participation; in turn, a lack of exercise can exacerbate insomnia symptoms (26). Therefore, a mutually reinforcing or mutually inhibiting developmental trajectory may emerge between PA and insomnia.

A bidirectional relationship between IGD and insomnia may form a self-perpetuating cycle. First, IGD may increase the risk of subsequent insomnia (29, 30). On the one hand, prolonged nighttime gaming exposure to blue light emitted by electronic screens can suppress melatonin secretion and disrupt circadian rhythms, thereby affecting sleep onset (31). On the other hand, the mechanisms driving this risk can be profoundly explained by the hyperarousal theory. According to this theory, insomnia is not merely a consequence of sleep deprivation, but rather a condition of persistent physiological and cognitive-emotional overactivation. During high-intensity gaming, the competitive and rewarding experiences continuously activate the sympathetic nervous system, keeping individuals in a state of heightened physiological arousal (32, 33). At the same time, chronic sleep deprivation weakens prefrontal executive function and self-control, reducing an individual’s ability to inhibit impulsive behavior and thereby increasing the risk of uncontrolled gaming (34). Therefore, insomnia may not only be a consequence of IGD but also a significant precursor, with the two potentially influencing each other over time.

Furthermore, physical activity, internet gaming disorder, and insomnia may not exist in isolation but are interconnected through a progressive, intrinsic relationship. Physical activity improves sleep not only through physiological mechanisms but also by altering digital behavior patterns: that is, physical activity provides an alternative reward, reducing compensatory dependence on online gaming and thereby decreasing the incidence of online gaming disorder; conversely, a reduction in the severity of online gaming disorder directly mitigates game-induced pre-sleep cognitive rumination, emotional arousal, and delayed sleep onset, ultimately alleviating insomnia. Thus, online gaming disorder may serve as a mediating factor between physical activity and insomnia.

Although the relationships among the aforementioned variables have received some empirical support, existing research still has several significant limitations. Most studies employ cross-sectional designs, making it difficult to determine the temporal sequence and causal direction among variables. Therefore, based on the aforementioned theoretical and empirical foundations, this study uses Chinese college students as participants and adopts a three-wave longitudinal design and a cross-lagged panel model (CLPM) to examine the bidirectional longitudinal relationships among PA, IGD, and insomnia symptoms, as well as their dynamic interaction mechanisms. The cross-lagged design allows identification of temporal sequences and predictive effects among variables while controlling for variable stability, thereby providing stronger evidence for understanding the underlying relationships among the three constructs. Based on this, this study proposes the following hypotheses:

H1: Physical activity and Internet gaming disorder are interrelated.

H2: Internet gaming disorder and insomnia are interrelated.

H3: Physical activity and insomnia are interrelated.

H4: Internet gaming disorder mediates the longitudinal relationship between physical activity and insomnia.

## 2 Methods

### 2.1 Participants and procedure

This study targeted undergraduate students enrolled at a comprehensive university in China and employed convenience sampling for data collection. The longitudinal survey consisted of three assessment time points, administered in March, June, and October 2025, with approximately three months between each consecutive assessment. This measurement interval was adopted to minimize participant fatigue from repeated measures and reduce sample attrition, thereby ensuring the integrity and quality of the longitudinal data. Prior to the assessment, all participants signed a written informed consent form and were explicitly informed that they could withdraw from the study at any time without conditions.

At the T1 stage, 673 questionnaires were distributed, with 593 valid responses collected, resulting in a valid response rate of 88.11%; at the T2 stage, 593 questionnaires were distributed to valid participants from T1, with 533 valid responses collected, resulting in a valid response rate of 89.88%; At the T3 stage, 533 questionnaires were distributed to valid T2 participants, with 497 valid responses collected, resulting in a valid response rate of 93.25%. Subsequently, data from the three waves were matched and integrated based on student ID numbers. Cases that did not fully participate in all three waves or exhibited obvious invalid response patterns (such as regular answering patterns or extreme consistency in responses) were excluded. Ultimately, 475 valid participants were included in the statistical analysis, resulting in a longitudinal sample retention rate of 80.10% (based on the valid T1 sample) and an overall valid response rate of 70.58%(based on the total number of questionnaires distributed at T1). The sample included 217 males (45.68%) and 258 females (54.32%), with an average age of 18.43 ± 1.71 years.

### 2.2 Instruments

#### 2.2.1 Physical activity

Physical activity levels were assessed using the Physical Activity Rating Scale-3 (PARS-3), a three-item self-report instrument designed to evaluate exercise intensity, duration, and frequency (35). Each dimension was scored on a 5-point scale, and the composite score was calculated using the following formula: exercise intensity × (exercise duration – 1) × exercise frequency. The total score ranged from 0 to 100 points. A higher total score indicates a higher level of physical activity participation. In this study, the Cronbach’s alpha coefficients for the three measurements of this scale were 0.74, 0.79, and 0.76, respectively.

#### 2.2.2 IGD symptoms

The nine-item Internet Gaming Disorder (IGD) checklist from the Diagnostic and Statistical Manual of Mental Disorders, Fifth Edition (DSM-5) was used to assess IGD symptoms (36). This scale evaluates the presence of core IGD symptoms over the past 12 months (answered as “yes” or “no”). The nine criteria include: preoccupation with gaming, withdrawal symptoms, tolerance, loss of control, prioritizing gaming over other activities, continuing to game despite negative consequences, lying about gaming time, escapism, and gaming-related functional impairment. The Chinese version of this checklist has been validated and demonstrates satisfactory psychometric properties (37). In this study, the Cronbach’s alpha coefficients for the three administrations of the scale were 0.81, 0.78, and 0.84, respectively.

#### 2.2.3 Insomnia

This study used the Athens Insomnia Scale (AIS) to assess the severity of insomnia among participants (38). This scale is a self-report instrument developed based on the diagnostic criteria for insomnia in the International Classification of Diseases, 10th Revision (ICD-10). It consists of 8 items designed to quantify the severity of insomnia symptoms. Each item uses a 0–3 Likert scale (0 = no problem, 3 = very severe), with a total score ranging from 0 to 24; a higher score indicates more severe insomnia symptoms. This scale has been validated in China and demonstrates good reliability and validity (39). In this study, the Cronbach’s alpha coefficients for the three measurements of this scale were 0.83, 0.87, and 0.92, respectively.

### 2.3 Statistics and analysis

All statistical analyses were conducted using SPSS 27.0 and Mplus 8.3. First, SPSS was used to perform descriptive statistical analyses of the main variables, including means, standard deviations, skewness, kurtosis, and correlation coefficients, to understand the distribution of PA, IGD, and insomnia symptoms across the three time points and their preliminary associations. Prior to formal model analysis, this study examined the internal consistency reliability of each scale at the three time points and reported Cronbach’s α coefficients. To investigate the temporal evolution and underlying mechanisms of physical activity, Internet gaming disorder, and insomnia, a three-phase cross-lagged panel model (CLPM) was constructed in Mplus 8.3, and maximum likelihood (ML) estimation was used to estimate parameters. In terms of model specification, the model included autoregressive paths to control for within-time-point stability and cross-lagged paths across time points, while allowing for within-time-point residual correlation, thereby ensuring the robustness of the estimation results. Model fit was evaluated using a comprehensive set of indices, including χ², the Comparative Fit Index (CFI), the Tucker-Lewis Index (TLI), the Root Mean Square Error of Approximation (RMSEA), and the Standardized Root Mean Square Residual (SRMR). Generally, CFI and TLI values greater than 0.90 indicate acceptable model fit, while values greater than 0.95 indicate good fit; RMSEA and SRMR values less than 0.08 indicate acceptable model fit, while values less than 0.05 indicate good fit. Furthermore, to examine the longitudinal bridging role of internet use disorders in the relationship between physical activity and insomnia, the study employed the bias-corrected Bootstrap method (with 5,000 repetitions) to test for indirect effects. If the 95% confidence interval does not include 0, the mediation is deemed to hold; this nonparametric testing method effectively circumvents the limitations of the normal-distribution assumption, thereby significantly enhancing the validity of the mediation test and the reliability of the conclusions.

## 3 Results

### 3.1 Descriptive statistics

**Table 1** presents the means and standard deviations of physical activity and online gaming disorder at different time points. At time point t1, the mean for physical activity was 14.23 with a standard deviation of 13.73; at time point t2, the mean was 14.08 with a standard deviation of 12.78; and at time point t3, the mean was 14.36 with a standard deviation of 12.47. For online gaming disorder, the mean at time point t1 was 1.47 with a standard deviation of 2.20; at time point t2, the mean was 1.51 with a standard deviation of 1.85; and at time point t3, the mean was 1.64 with a standard deviation of 1.97. The mean score for insomnia at time point t1 was 5.73, with a standard deviation of 4.29; at time point t2, the mean was 6.04, with a standard deviation of 4.39; and at time point t3, the mean was 5.60, with a standard deviation of 4.13.

**Table 1:** Descriptive statistics of each variable at different time points.

Variable	Mean ± standard deviation	Skewness	Kurtosis
PA (T1)	14.23 ± 13.73	2.31	8.34
PA (T2)	14.08 ± 12.78	1.75	5.65
PA (T3)	14.36 ± 12.47	1.56	5.43
IGD (T1)	1.47 ± 2.20	1.90	3.23
IGD (T2)	1.51 ± 1.85	1.53	2.59
IGD (T3)	1.64 ± 1.97	1.4	1.91
INS (T1)	5.73 ± 4.29	0.71	0.38
INS (T2)	6.04 ± 4.39	0.83	0.82
INS (T3)	5.60 ± 4.13	0.86	1.09

PA, Physical Activity; IGD, Internet Gaming Disorder; INS, Insomnia; T1, T2, and T3 represent the three time points, respectively..

### 3.2 Correlation analysis

The correlation coefficients of each variable at different time points are shown in **Table 2**. The results indicate that PA, IGD, and INS all exhibit significant autocorrelation (r = 0.334–0.546), demonstrating good stability across time. Regarding cross-temporal correlations, IGD and INS were significantly positively correlated at all time points (r = 0.190–0.345), while PA was significantly negatively correlated with both IGD and INS (r = -0.156–0.289).

**Table 2:** Correlation analysis results.

Variable	PA (T1)	PA (T2)	PA (T3)	IGD (T1)	IGD (T2)	IGD (T3)	INS (T1)	INS (T2)	INS (T3)
PA (T1)	--								
PA (T2)	0.537***	--							
PA (T3)	0.346***	0.460***	--						
IGD (T1)	-0.285***	-0.208***	-0.188***	--					
IGD (T2)	-0.285***	-0.289***	-0.262***	0.546***	--				
IGD (T3)	-0.218***	-0.212***	-0.273***	0.334***	0.411***	--			
INS (T1)	-0.285***	-0.194***	-0.156***	0.327***	0.318***	0.205***	--		
INS (T2)	-0.257***	-0.288***	-0.224***	0.298***	0.345***	0.248***	0.496***	--	
INS (T3)	-0.262***	-0.260***	-0.273***	0.190***	0.321***	0.333***	0.377***	0.531***	--

***p <.001.

### 3.3 CLPM results

The correlation coefficients of each variable at different time points are shown in **Table 3**. The results indicate that PA, IGD, and INS all exhibit significant autocorrelation (r = .334–.546), demonstrating good stability across time. Regarding cross-temporal correlations, IGD and INS were significantly positively correlated at all time points (r = .190–.345), while PA was significantly negatively correlated with both IGD and INS (r = -.156–.289).

**Table 3:** Cross-lagged model fit indices.

Model fit indices	χ²	df	CFI	TLI	RMSEA	SRMR
Value	27.118	9	0.983	0.933	0.065	0.024

**Figure 1:**
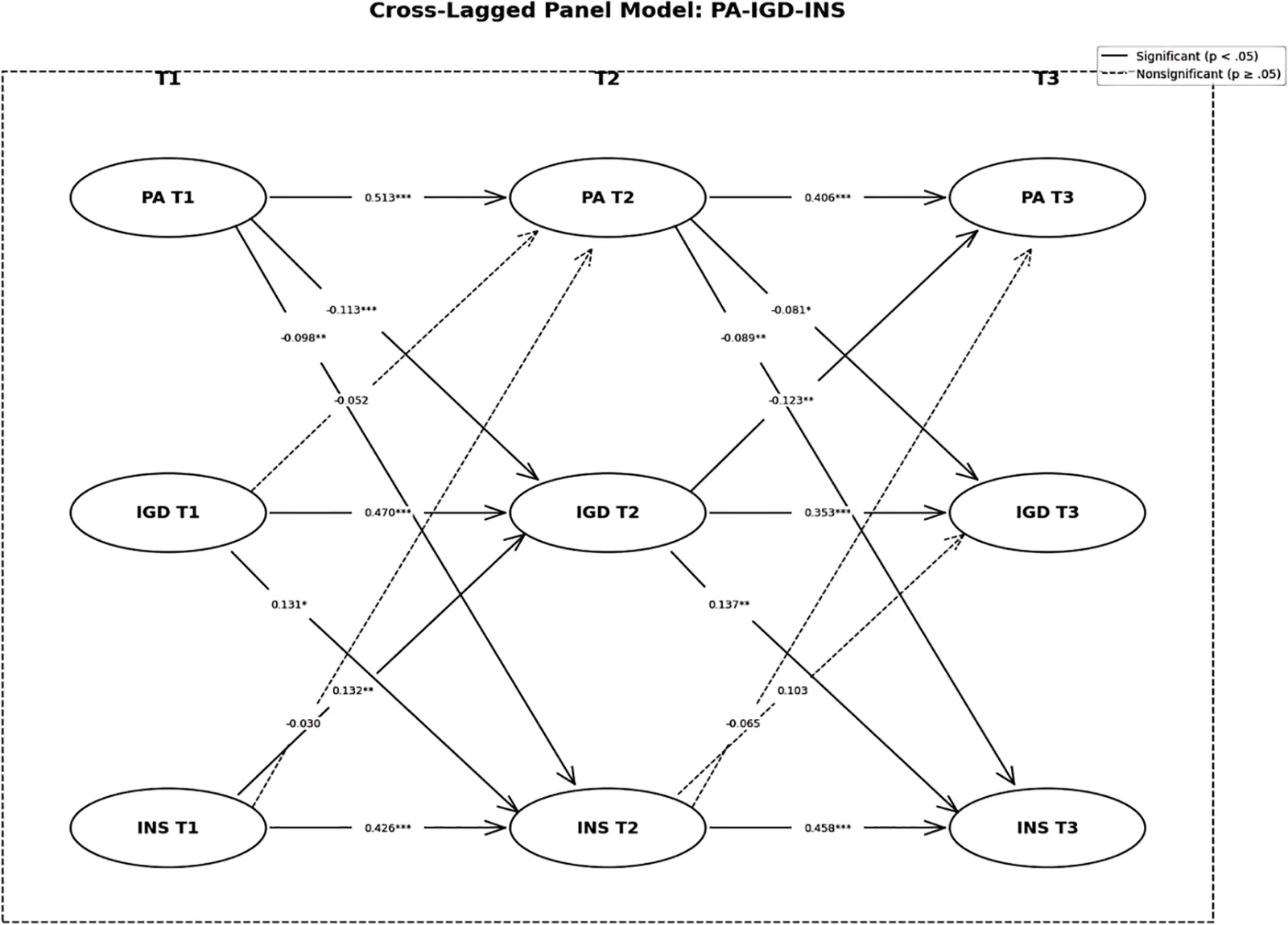
Cross-lagged panel results of physical activity, internet gaming disorder, and insomnia. *p<.05, **p<.01, ***p<.001. PA, Physical Activity; IGD, Internet Gaming Disorder; INS, Insomnia.

As shown in **Figure 1**, after controlling for the autoregressive effects of the variables, all variables exhibited significant cross-temporal stability. PA, IGD, and INS at T1 all significantly and positively predicted their own levels at T2 (with β values of 0.513, 0.470, and 0.426, respectively, and 95% CIs of [0.418, 0.598],[0.370, 0.558], and [0.351, 0.499], respectively), and PA, IGD, and INS at T2 also significantly and positively predicted their own levels at T3 (β = 0.406, 0.353, and 0.458, respectively; 95% CIs = [0.315, 0.504],[0.243, 0.458], and [0.365, 0.537], respectively). Cross-lagged effects indicated that the role of physical activity as a protective precursor was robust; PA at both T1 and T2 significantly and negatively predicted subsequent IGD (T1 PA → T2 IGD: β = -0.113, 95% CI [-0.174, -0.052]; T2 PA → T3 IGD: β = −0.081, 95% CI [−0.153, −0.007]) and INS (T1 PA → T2 INS: β = −0.098, 95% CI [−0.159, −0.034]; T2 PA → T3 INS: β = -0.089, 95% CI [-0.144, -0.029]). Internet gaming disorder exhibited a significant risk-increasing effect on subsequent insomnia; IGD at both T1 and T2 significantly and positively predicted subsequent INS (T1 IGD → T2 INS: β = 0.131, 95% CI [0.031, 0.230]; T2 IGD → T3 INS: β = 0.137, 95% CI [0.042, 0.232]); simultaneously, IGD exhibited negative feedback inhibition on subsequent physical activity, with T2 IGD significantly negatively predicting T3 PA (β = -0.123, 95% CI [-0.196, -0.033]), whereas the predictive effect of T1 IGD on T2 PA was not significant (β = -0.052, p = 0.175).Insomnia did not significantly predict subsequent physical activity (T1 INS → T2 PA: β = -0.030, p = 0.408; T2 INS → T3 PA: β = -0.065, p = .151). However, T1 INS significantly and positively predicted T2 IGD (β = 0.132, 95% CI [0.055, 0.209]), while the predictive effect of T2 INS on T3 IGD was not significant (β = 0.103, p = .055).

Mediation analysis based on 5,000 bias-corrected bootstrap samples indicated a significant longitudinal mediating effect of T1 PA on T3 INS via T2 IGD (effect size = -0.005,95% CI [−0.010, −0.002]), and the direct effect of T1 PA on T3 INS was also significant (effect size = −0.029,95% CI [−0.048, −0.010]). The total effect was −0.037 (95% CI [−0.059, −0.018]), indicating that physical activity not only directly improves subsequent insomnia but also exerts an indirect protective effect by reducing the level of Internet gaming disorder.

## 4 Discussion

Based on three waves of longitudinal data from Chinese college students, this study employed a cross-lagged panel model to systematically examine the dynamic relationships among physical activity (PA), Internet gaming disorder (IGD), and insomnia (INS). The results indicate that physical activity consistently and negatively predicts subsequent levels of Internet gaming disorder and insomnia; Internet gaming disorder consistently and positively predicts subsequent insomnia; and insomnia only and positively predicts subsequent Internet gaming disorder in the early stages, while its predictive effect on physical activity did not reach statistical significance. Furthermore, IGD plays a significant longitudinal mediating role between physical activity and insomnia. Overall, this study not only validates the long-term associations among these three variables but also reveals a potential mechanism by which physical activity alleviates insomnia symptoms by reducing the risk of IGD, providing new longitudinal evidence for understanding the dynamic, interactive processes between health behaviors and sleep health among college students.

First, this study found that physical activity consistently predicted lower subsequent levels of IGD among college students. This finding aligns with the growing body of longitudinal evidence suggesting a protective effect of physical activity on gaming behaviors. For instance, a six-month longitudinal study by Huang et al. (2022) observed temporal associations between physical activity and problematic internet use (40), while a Swiss male cohort study identified physical activity as a predictor of IGD progression (41). Our study extends these findings by demonstrating this relationship within a Chinese college student sample and over a shorter time frame. However, unlike Hu et al. (2026), who found reciprocal within-person dynamics between IGD symptoms and physical activity among adolescent gamers (42), we observed that physical activity predicted lower subsequent IGD across both intervals (T1→T2 and T2→T3), with a significant reciprocal effect only emerging from IGD to physical activity in the later interval (T2→T3). This relationship can also be explained by the alternative reward theory: when individuals obtain positive reinforcement and fulfill psychological needs through real-world activities, their compensatory reliance on instant online rewards decreases (43, 44). Physical activity not only improves mood but also provides real-world positive resources such as a sense of achievement and social interaction, which precisely meet these psychological needs. From the perspective of behavioral addiction development mechanisms, this aligns with the I-PACE model (45). For college students undergoing psychosocial transition, who face multiple pressures such as academic competition, interpersonal adaptation, and future planning, and whose prefrontal executive control functions are not yet fully developed, they are more likely to turn to online gaming as a compensatory coping strategy driven by negative emotions. Physical activity can improve emotional regulation, enhance self-control, and reduce negative emotional experiences (46). Consequently, it may help interrupt the reinforcement of the “emotion-cognition-behavior” cycle in the addiction process, potentially lowering the risk of gaming addiction.

This study also found that IGD negatively predicted physical activity in the later stage (i.e., T2 IGD significantly associated with lower T3 PA), indicating a dynamic but asymmetric interaction between the two. Compared with the protective effect of physical activity on IGD in earlier stages, the impact of IGD on physical activity shows a lag. This suggests that the association between online gaming behavior and the erosion of daily lifestyles may require a certain period of accumulation before it gradually becomes apparent. According to the theory of behavioral displacement, individuals have limited daily discretionary time and energy. When online gaming occupies an increasing share of leisure time, real-world activities, such as physical activity, are often the first to be displaced (47). At the same time, prolonged immersion in gaming tends to foster sedentary patterns and weaken motivation to exercise (18, 19). Therefore, for college students with long-standing Internet Gaming Disorder, the objective, ongoing displacement of their leisure time and the subjective, progressively weakening motivation to exercise may jointly contribute to lower their physical activity levels over time.

Second, this study found that physical activity consistently predicted lower subsequent insomnia levels, consistent with findings from previous studies (48, 49). Mechanistically, physical activity may improve sleep primarily through physiological and circadian pathways: On the one hand, physical activity increases energy expenditure and elevates the body’s need for sleep homeostasis, thereby strengthening the drive for sleep at night. For college students, who commonly engage in sedentary behaviors, participating in physical activity significantly increases daytime energy expenditure, effectively alleviating sleep onset difficulties caused by a lack of physical exertion; On the other hand, regular physical activity may enhance circadian rhythm signals and stabilize the sleep-wake cycle, which is particularly important for college students who commonly experience irregular schedules and sedentary behaviors (50). It is worth noting that the negative predictive effect of physical activity on insomnia remained significant across both time points in this study, indicating that this association is sustained rather than short-term. This suggests that regular physical activity is not only associated with better current sleep but may also predict a long-term protective association with future sleep health among college students by promoting the adoption of a healthy lifestyle, further supporting its practical value as a key behavioral strategy for insomnia prevention and health promotion.

Contrary to the research hypothesis, this study did not find a significant predictive effect of insomnia on subsequent physical activity. Although previous studies have suggested that long-term sleep disturbances are associated with increased fatigue, decreased energy, and reduced motivation to exercise, which may in turn be linked to lower participation in physical activity (51, 52), this effect was not supported in the present study. One possible explanation is that individual subjective states do not entirely determine physical activity behavior among college students but is also influenced by external factors such as course schedules, the campus environment, and social activities. Even with some degree of sleep problems, individuals may still maintain a basic level of exercise participation. Furthermore, this study measured overall physical activity levels rather than exercise persistence or motivation; therefore, the short-term fatigue associated with insomnia may not be sufficient to translate into observable long-term changes in overall physical activity levels.

Third, this study found a bidirectional relationship between IGD and insomnia, but this bidirectional effect is not entirely symmetrical. On the one hand, Internet Gaming Disorder consistently predicted subsequent insomnia across both time intervals; on the other hand, insomnia significantly predicted subsequent IGD only from T1 to T2, while this effect was not statistically significant from T2 to T3. The sustained predictive role of IGD on insomnia is consistent with previous research findings (53), further supporting the applicability of the hyperarousal theory in the field of behavioral addiction. College students have relatively flexible nighttime schedules, and the impact of gaming behavior on sleep is often explained either by nighttime gaming directly occupying sleep time or by blue light exposure from electronic devices before bed inhibiting melatonin secretion (54). Additionally, sustained cognitive activity and emotional arousal before bedtime are important mechanisms underlying the development of insomnia. Individuals with online gaming disorder often exhibit higher levels of reward sensitivity, psychological preoccupation, and gaming cravings; even after stopping play, they may continue to think about game content, anticipate reward feedback, or repeatedly replay the gaming process in their minds. This persistent state of cognitive arousal hinders the body’s ability to transition smoothly from wakefulness to sleep, thereby increasing the risk of difficulty falling asleep and sleep maintenance disorders (32). Therefore, Internet Gaming Disorder is not merely a concomitant phenomenon of sleep problems but may also serve as a key behavioral mechanism driving the progression of insomnia.

Unlike the persistent impact of IGD on insomnia, the predictive role of insomnia on IGD is limited to the early stages. This finding aligns with recent longitudinal studies suggesting that sleep problems may precede the onset of behavioral addiction (34). Sleep deprivation impairs prefrontal executive function and self-control (55, 56), increasing an individual’s tendency toward impulsive decision-making and the generation of negative emotions, making them more likely to rely on activities offering immediate rewards to alleviate fatigue and negative emotions. Among college students, online gaming—due to its high accessibility, immediate feedback, and emotional compensation functions—often serves as a key coping mechanism for individuals with poor sleep (57). However, this study did not find this effect to persist in subsequent stages, which may suggest that the influence of insomnia on IGD is more pronounced during the early stages of behavioral addiction formation; once gaming behavior becomes established, its development may be driven more by habituation mechanisms and the reward system.

Fourth, the study further found that Internet Gaming Disorder plays a significant longitudinal mediating role between physical activity and insomnia. Specifically, T1 PA predicted T3 insomnia through T2 IGD. Mechanistically, physical activity not only improves an individual’s physiological state but also reduces compensatory reliance on online gaming by providing rewarding experiences and opportunities for real-life social interaction. When individuals receive more positive reinforcement from their real-life environment, the importance of online gaming as a tool for emotional regulation diminishes, thereby reducing game-related cognitive preoccupation and emotional arousal levels, and ultimately improving sleep quality.

Theoretically, this study provides new longitudinal evidence for examining pairwise relationships; by utilizing three waves of longitudinal data, it reveals the longitudinal associations among physical activity, Internet gaming disorder, and insomnia, thereby offering more robust evidence for inferring causal relationships between health behaviors and sleep problems; at the same time, by integrating these three factors into a single model, this study clarifies the bridging role of Internet gaming disorder, providing supplementary longitudinal evidence. In practice, physical activity can serve as a low-cost, multi-benefit intervention strategy: universities should not only enhance college students’ physical activity levels and reduce the risk of gaming addiction by optimizing exercise environments and curricula, but also prioritize controlling online gaming disorder as a key entry point for improving insomnia when implementing sleep health promotion initiatives. However, given the modest effect sizes and methodological constraints, these implications should be considered preliminary and require validation through randomized controlled trials with extended follow-up periods.

Although this study provides supplementary longitudinal evidence, several limitations remain. First, the study sample was drawn exclusively from Chinese college students; therefore, the applicability of the findings to other age groups and cultural contexts requires further validation. Second, this study relied on self-report measures for relevant variables; future research could validate these findings by integrating objective physical activity and sleep monitoring, as well as digital behavior log data. Third, this study did not further distinguish among different types of exercise, exercise intensities, or types of online games, yet these factors may exert varying effects. Future research could further explore the differential roles of different forms of exercise in improving online gaming disorder and sleep problems, and validate their potential mechanisms through randomized controlled intervention studies. Finally, the traditional CLPM failed to disaggregate between-person from within-person effects, and future research could employ the RI-CLPM to separate them; the three-month measurement intervals may not be optimal; and the study relied on self-report data.

## 5 Conclusion

Based on three waves of longitudinal data from Chinese college students, this study found that physical activity can consistently reduce levels of internet gaming disorder and insomnia, while internet gaming disorder increases the risk of subsequent insomnia. Further analysis indicates that online gaming disorder plays a significant longitudinal mediating role between physical activity and insomnia. The findings reveal the dynamic interactive mechanisms among physical activity, online gaming disorder, and insomnia, providing new theoretical evidence for understanding the behavioral foundations of sleep health in college students and offering important practical insights for physical activity-based insomnia prevention and intervention strategies.

## Data Availability

The original contributions presented in the study are included in the article/supplementary material. Further inquiries can be directed to the corresponding author.
