# Correction: Longitudinal relationships among physical activity, internet gaming disorder, and insomnia: a cross-lagged panel analysis

**DOI:** 10.3389/fpsyt.2026.1938472

**Published:** 2026-08-05

**Authors:** Huijie Wang, Tiantian Cao, Lin Luo

**Affiliations:** 1 School of Physical Education, Jiangxi Normal University, Nanchang, Jiangxi, China; 2 Shangrao High Speed Railway Economic Experimental Zone Middle School, Shangrao, Jiangxi, China

**Keywords:** cross-lagged panel model, insomnia, internet gaming disorder, longitudinal mediation effect, physical activity

Affiliation “Shangrao High Speed Railway Economic Experimental Zone Middle School, Shangrao, Jiangxi, China” was omitted from the paper.

This affiliation has now been added to author “Tiantian Cao”.

The original version of this article has been updated.

